# Investigation of alpha-synuclein in patients with late-onset schizophrenia

**DOI:** 10.1192/j.eurpsy.2022.808

**Published:** 2022-09-01

**Authors:** E. Palchikova, M. Nikolaev, A. Lavrinova, N. Neznanov, S. Pchelina, N. Zalutskaya

**Affiliations:** 1 V.M.Bekhterev National Medical Research Center for Psychiatry and Neurology, Geriatric Psychiatry, St.Peterburg, Russian Federation; 2 Saint Petersburg Nuclear Physics Institute Named by B.P. Konstantinovof National Research Centre“Kurchatov Institute”, Molecular Biology, Saint Petersburg, Russian Federation; 3 Bekhterev National Medical Center for Psychiatry and Neurology, Geriatric Psychiatry, Saint-Petersburg, Russian Federation

**Keywords:** schizophrénia, proteostasis, alpha-synuclein, late-onset psychosis

## Abstract

**Introduction:**

There is no consensus about whether late-onset schizophrenia(LOS) is a type of schizophrenia or a secondary psychotic disorder. One of the theories of the occurrence of late-onset psychoses is neurodegeneration caused by the imbalance of proteostasis.

**Objectives:**

To study the concentration and expression of alpha-synuclein in patients with LOS compared with controls.

**Methods:**

The study involved 42 patients with the ICD-10 criteria of schizophrenia with the onset of the disease after 45 years and 104 controls with no dementia and severe somatic pathology, comparable in age and gender. The alpha-synuclein level was estimated in a lymphocytic cell fraction from patients with LOS N=42 and controls N=104 using the Human alpha-synuclein ELISA kit. The expression of the SNCA gene was studied in 22 LOS patients and 22 controls and determined by PCR using the SYBR Green Supermix kit and the CFX96 instrument in comparison with the referent genes using previously developed primers. LOS group underwent psychopathological examination including scales. Statistics: SPSS 12.0; the Mann–Whitney test; exponential regression analysis; data is given as median (min–max). The level of significance was set at p<0.05.

**Results:**

The alpha-synuclein level is higher in patients with LOS (9.21 (0.78-29.52)) compared to control (6,355 (0.46-35.44)), p=0.024. The mRNA level of the SNCA gene is higher in the LOS group (0.533 (0.089-1.406)) compared to controls (0.087 (0.016-0.266) , p<0.001.

**Conclusions:**

Our study shows the relationship of alpha-synuclein with the manifestations of LOS. Obtained novel data can open up new targets for therapy and bring it closer to understanding the phenomenon.

**Disclosure:**

No significant relationships.

